# Immersive VR and Education: Embodied Design Principles That Include Gesture and Hand Controls

**DOI:** 10.3389/frobt.2018.00081

**Published:** 2018-07-24

**Authors:** Mina C. Johnson-Glenberg

**Affiliations:** Department of Psychology, Arizona State University, Tempe, AZ, United States

**Keywords:** immersive virtual reality, embodiment, gesture, stem education, mixed reality, VR, educational design, XR

## Abstract

This article explores relevant applications of educational theory for the design of immersive virtual reality (VR). Two unique attributes associated with VR position the technology to positively affect education: (1) the sense of presence, and (2) the embodied affordances of gesture and manipulation in the 3rd dimension. These are referred to as the two profound affordances of VR. The primary focus of this article is on the embodiment afforded by gesture in 3D for learning. The new generation of hand controllers induces embodiment and agency via meaningful and congruent movements with the content to be learned. Several examples of gesture-rich lessons are presented. The final section includes an extensive set of design principles for immersive VR in education, and finishes with the *Necessary Nine* which are hypothesized to optimize the pedagogy within a lesson.

“Movement, or physical activity, is thus an essential factor in intellectual growth, which depends upon the impressions received from outside. Through movement we come in contact with external reality, and it is through these contacts that we eventually acquire even abstract ideas.”

(Montessori, 1966)

## The two profound affordances

In the early 1930's, Dr. Montessori understood that learning relied on how our physical bodies interacted with the environment. For her, the environment was physical. Today, we are able to digitize our environments and the affordances approach infinity. For several decades, the primary interface in educational technology has been the mouse and keyboard; however, those are not highly embodied interface tools (Johnson-Glenberg et al., 2014a). Embodied, for the purposes of education, means that the learner has initiated a physical gesture or movement that is well-mapped to the content to be learned. As an example, imagine a lesson on gears and mechanical advantage. If the student is tapping the *s* on the keyboard to make the gear spin that would be considered less embodied than spinning a fingertip on a screen to manipulate a gear with a synchronized velocity. With the advent of more natural user interfaces (NUI), the entire *feel* of digitized educational content is poised to change. Highly immersive virtual environments that can be manipulated with hand controls will affect how content is encoded and retained. One of the tenets of the author's Embodied Games lab is that doing actual physical gestures in a virtual environment should have positive, and lasting, effects on learning in the real world. Tremendous opportunities for learning are associated with VR (Bailenson, 2017) and one of the most exciting aspects of VR “is its ability to leverage interactivity” (Bailenson et al., 2008).

Immersive and interactive VR is in its early days of educational adoption. It will not prove to be a panacea for every disengaged student (as is sometimes stated in the popular press), nor do we expect future scholars to spend entire days in virtual classrooms (see fiction by Cline, 2011). However, now that some of VR's affordability and sensorial quality issues are being addressed, it is reasonable to believe that VR experiences will become more ubiquitous in educational settings. When the demand comes, the community should be ready with quality educational content. There are few guidelines now for how to make optimal educational content in VR, thus this theory article ends with several concrete design principles.

Two attributes of VR may account for its future contributions to education. These we call the two *profound affordances*. The first profound affordance is the **feeling of presence** which designers must learn to support, while not overwhelming learners. The sense of presence is fairly well understood at this point. Slater and Wilbur (1997) describe it as the feeling of being there. The second profound affordance pertains to **embodiment and the subsequent agency associated with manipulating content** in three dimensions. Manipulating objects in three dimensional space gives a learner unprecedented personal control (agency) over the learning environment. This article focuses on how gesture and the use of hand controls can increase agency and learning. The basis for this prediction is the research on embodiment and grounded cognition (Barsalou, 2008). Although other methods for activating agency can be designed into VR learning environments (e.g., using eye gaze and/or speech commands), it may be the case that gesture plays a special role. Gesture kinesthetically activates larger portions of the sensori-motor system and motoric pre-planning pathways than the other two systems and gesture may lead to stronger memory traces (Goldin-Meadow, 2011). Another positive attribute of engaging the learner's motoric system via the hand is that it is associated with a reduction in simulator sickness (Stanney and Hash, 1998)^1^.

VR for education should take full advantage of 3D object manipulation using the latest versions of handheld controllers (as well as, gloves and in-camera sensors to detect joints, etc.). Gathering and analyzing gestures in 3D is an area in need of more research and evidence-based design guidelines (Laviola et al., 2017). Because randomized control trials (RCT) are just starting to be published on immersive VR in education, this article is primarily theory-based. The goal of this article is to share some of what has been learned about embodiment in mixed reality platforms for education, and to produce a set of design principles for VR in education to assist this nascent field as it matures.

### Vocab lesson: VR, presence, and agency

In this article, the term VR refers to an immersive experience, usually inside a headset where the real world is not seen for 360°. (We do not focus on CAVES as the cost precludes large scale adoption in the K-16 education arena. In addition, it is probably more embodied to see virtualized body parts, as is common in headset experiences.) In VR, the learners can turn and move as they do in the real world, and the digital setting responds to the learner's movements. *Immersive VR* systematically maintains an illusion of presence, such that learners feel their bodies are inside the virtual environment. Being able to see evidence of the real world, even in the periphery, would mean the platform should be deemed augmented or mixed reality (AR/MR). A three dimensional object or avatar moving on a regular-sized computer monitor is never “VR”; we hope that educators soon stop conflating the terms and phenomena.

The term presence is also defined in the glossary of a recent book dedicated to VR and education (Dede and Richards, 2017). Presence is a “particular form of psychological immersion, the feeling that you are at a location in the virtual world.” The sensations are reported to be quite visceral. It is true that the sensation of being on location and unmindful of real world cues can occur even when users interact with “low immersion environments” (e.g., on a smartphone), but the content must be extremely engaging. In a full immersion headset experience, the feeling of being in a different location is systematic and usually instantaneous. The presence associated with VR is one of the most immediate and best documented phenomena. Thus, presence is deemed the first profound affordance of VR. Several surveys are available for assessing the amount of presence in a mediated experience (Slater and Wilbur, 1997; Makransky et al., 2017). Slater's lab has led extensive research on presence and his group has also pioneered a method for assessing presence without the use of surveys (Bergstrom et al., 2017).

Immersive VR has the ability to immediately transport the user to a limbically heightened emotional space that can have positive effects on attention and engagement; this is one reason why educators believe that learning will be positively affected. The *Google Expeditions* series relies on presence to immediately engage learners. A recent exploratory study explicitly states that the presence afforded by the 3D technology “opens up” the senses and mind for learning (Minocha et al., 2017). Minocha et al. further hypothesize that because the students are in control of where they look and for how long, they can then follow “… their interest and curiosity, hence giving them a sense of control and empowerment over their own exploration.” Whenever users feel they have control over the environment, they experience *agency*.

#### The second profound affordance

Is it the case that learning in 3D is always better than in 2D? Will the learner acquire knowledge faster and show better retention? This is a vital question that deserves further research. Jacobson (2011) believes the answer is yes, at least when the learning relates to skill acquisition. For example, middle school students recalled more declarative knowledge, i.e., symbols and spatial layout, after experiencing a three-dimensional ancient Egyptian temple presented in a dome environment, compared to a desktop version with the same information (Jacobson, 2011). However, the multi-user dome and the *Expeditions Cardboard* experiences are examples of virtual environments where hand controls and gesture were not used. The ability to control movement via gaze is one form of agency, but the ability to control and manipulate objects in the 3D environment is perhaps a different and deeper form of agency with many more degrees of freedom. The hypothesis is, the more agentic the learning, the better. Here we use the term agency to connote the user has individual (self-initiated) control and volition over the individual virtual objects in the environment. In the educational field, this definition of agency would reside under “self-directed constructs” in the Snow et al. (1996) provisional taxonomy of conative constructs in education.

The idea of agency is baked into the second profound component of VR. The newest generations of VR includes synced hand controls, so that gesture and manipulating objects in VR with an NUI keeps becoming more affordable. Our prediction is that hand controls will have long lasting effects on the types of content and the quality of the pedagogy that can be designed into educational spaces. Jang et al. (2016) utilized a yoked-pair design, such that one participant manipulated a virtualized 3D model of the inner ear, while another participant viewed a recording of the interaction. Results indicate that participants in the manipulation group showed greater posttest knowledge (via drawing) than the group that observed the manipulations. The manipulation (with a joystick-like device) is a form of gestural control that affords agency, and to understand how these constructs interact a clearer definition of embodiment is in order. Our research communities are in the early stages of exploring the affordances of VR and principles for design in education are also needed.

### Embodiment

Proponents of embodiment hold that the mind and the body are inextricably linked (Wilson, 2002). A compelling example of how the body's actions give rise to meaning comes from Hauk et al. (2004); they used functional magnetic resonance imaging (fMRI) to measure activation in regions of interest as participants listened to action verbs such as *lick, pick*, and *kick*. The researchers observed significantly more somatotopic activation of the premotor and motor cortical systems that specifically control the mouth, the hands, and the legs (respectively). These overlearned words, which were first experienced and mapped to meaning in childhood, are still activating specific motor areas in the adult brain. This is intriguing because it suggests that active, motor-driven concepts may stimulate distributed semantic networks (meaning), as well as the associated motor cortices which would have been used to learn long ago, in childhood. Semantics is part of an active learning system in humans. The way human and environmental systems work together to navigate the world is also termed “enactive cognitive science” by Varela et al. (1991; revised 2016). Varela et al. offer an eloquent description of how cognition can be viewed as an “interconnected system of multiple levels of sensori-motor subnetworks” (p. 206)^2^.

Embodied learning theory has much to offer designers of VR content, especially when the hand controls are used. The strong stance on embodiment and education holds that the body should be moving, not just reading or imaging, for a high level of embodiment to be in a lesson (Johnson-Glenberg, 2017; Johnson-Glenberg and Megowan-Romanowicz, 2017). When a motoric modality is added to the learning signal, more neural pathways will be activated and this may result in a stronger learning signal or memory trace. Several researchers posit that incorporating gesture into the act of learning should strengthen memory traces (Broaders et al., 2007; Goldin-Meadow, 2011). It may be the case that adding more modalities to the act of learning (beyond the usual visual and auditory ones) will continue to increase the strength of the memory trace. The modality of interest in this article is gesture. This article uses the term gesture to mean both the movement as a communicative form and the action used to manipulate virtual objects in the VR environment. The gesture-enhancing-the-memory trace argument can also be framed as one of levels of processing, which is a well-studied concept in cognitive psychology (Craik and Lockhart, 1972). The concept of “learning by doing” is also relevant to this article and is supported by the self-performed task literature in the psychology arena (see Engelkamp and Zimmer, 1994). They found that when participants *performed* short tasks, the task-associated words were better remembered compared to conditions where the participants read the words, or saw others perform the tasks.

Research on non-mediated forms of gesture in the educational arena has been fruitful. As an example, when teachers gesture during instruction, students retain and generalize more of what they have been taught (Goldin-Meadow, 2014). Recently, Congdon et al. (2017) showed that simultaneous presentation of speech and gesture in math instruction supports generalization and retention. Goldin-Meadow (2011) posits that gesturing may “lighten the burden on the verbal store” in a speaker's mind, and that gesturing may serve to offload cognition (Cook and Goldin-Meadow, 2006). Research supports that gestures may aid learners because learners use their own bodies to create an enriched representation of a problem grounded in physical metaphors (see Hostetter and Alibali, 2008; Alibali and Nathan, 2012; Nathan et al., 2014).

Several researchers also highlight that the gesture, or movement, should be congruent to the content being learned (Segal et al., 2010; Black et al., 2012). That is, the gesture should map to the instructed concept. For example, if the student is learning about the direction and speed of a spinning gear, then it would be important for the student's spinning hand gesture to go in the same direction and near the same speed as the virtual gear on screen (Johnson-Glenberg et al., 2015). An example of a low congruence gesture would be a “push forward” gesture to start a gear train spinning (the “push” is a default gesture for the *Kinect*™ sensor). Glenberg and Kaschak (2002) explore the effects of gesture and embodiment by varying the direction of button pushes in a sentence sensibility judgment task. If the button push action was away from the body and the sentence text was congruent to motion (i.e., “Give the pencil to X.”), then the reaction time to judge sensibility was significantly faster. Action congruent sentences were judged faster than the action incongruent sentences. As a final example of the importance of congruence, in a study by Koch et al. (2011), participants reacted faster in a Stroop task when using congruent gestures. The congruent gestures involved making an up movement attached to a word like “happy,” compared to the more incongruent downward gesture.

One hypothesis is that when learners are activating congruent and associated sensori-motor areas, they may learn the content faster and in a deeper manner. Gestures may provide an additional code for memory (again, strengthening the trace) as well as adding additional retrieval cues. Learners with stronger memory traces should do better on post-intervention tests. Work in the physics education domain supports the hypothesis that being active and engaging the body during encoding positively affects learning. In a recent Kontra et al. study, participants were randomly assigned to one of two roles in a learning dyad, either active or observant (Kontra et al., 2015). Participants who were active and physically held bicycle wheels spinning on an axle learned more about angular momentum compared to those who observed the spinning wheels. In an extension of Kontra's lab study, fMRI revealed that the level of the BOLD signal in the brain motor regions of interest (left M1/S1) significantly predicted content knowledge test performance (Kontra et al., 2015). The study of effects of activating motoric regions via gesture is a field of great interest for embodiment researchers and educators.

With the advent of VR hand controls, where human hand gestures can be transformed into near-infinite outcomes, it would be helpful to have a set of best practices for creating gesture-based educational VR content. Recently, it appears the term “embodiment” is being used in the VR research field to mean “a perceptual illusion, …the body ownership illusion” referring to one's avatar on screen (Bertrand et al., 2018). If this broad, human-to-avatar–body-swap definition of embodiment takes hold, then perhaps gesture would be considered a sub-type of VR embodiment. It remains to be seen how the term will evolve, but clearly a taxonomy would be helpful.

### Taxonomy of embodiment for education in VR

As with all theories, there are inclusive (weak) ones that start the spectrum, and exclusive (strong) ones that end it. One inclusive theoretical stance on embodied learning would be that any concept that activates perceptual symbols (Barsalou, 1999) is by its nature embodied. Following this stance, all cognition is embodied because early, original knowledge is gained via the body and its interactions with the environment, even new concepts that are later imagined. The environment's affordances (Gibson, 1979) shape and constrain how our bodies interact, ergo, cognition continues to be formed and expanded by these interactions. In an inclusive interpretation, according to some researchers, cognition would be broadly defined to include all sensory systems and emotions (Glenberg, 2010; Glenberg et al., 2013). A more exclusionary stance is one that distinguishes between low and high levels of embodiment. For a lesson to be deemed highly embodied, the learner would need to be *physically active;* the learner would have to kinesthetically activate motor neurons. Some principles for designing embodied education into MR platforms have been suggested (Lindgren and Johnson-Glenberg, 2013), and AR design principles have been proposed (Dunleavy, 2014); however, there are no design guidelines for VR that are based on embodiment. Given the new affordances of VR hand controls, it seems time to reframe some of this lab's previous embodied principles.

A more exclusionary definition of embodiment for education was proposed by this lab in 2014 (Johnson-Glenberg et al., 2014a) and updated recently (Johnson-Glenberg and Megowan-Romanowicz, 2017). That taxonomy posited four degrees of embodiment based on three constructs: (a) amount of sensori-motor engagement, (b) how congruent the gestures were to the content to be learned, and (c) amount of “immersion” experienced by the user. Each construct will be expanded upon.

#### Sensori-motor engagement

In terms of sensori-motor engagement via gesture (construct a), the first distinction relates to the magnitude of the motor signal. This means that walking or large arm movements activate more sensori-motor neurons than standing or swiping a finger across a screen. The magnitude of the movement should probably be part of the metric, but it is perhaps less important than whether the gesture is well-matched to the content to be learned (construct b). A small, yet highly congruent movement may be just as effective as a large one that is only loosely related to the learning concept. That is an experiment that needs to be conducted.

#### Congruency of the gesture

Construct b refers to the congruency of the gesture, that is, the movement should be well-mapped to the concept to be learned. The gesture should support the gist of the content and give meaningful practice to the learning goal; however, the movement need not be a perfect isomorphic match. In the spinning gears example, a mediated lesson was created to instruct in mechanical advantage for gear systems (Johnson-Glenberg et al., 2015). The *Microsoft Kinect* sensor was used to capture the direction and speed of the spin of the learner's arm. The learner extended his/her arm in front of the body and rotated it around the shoulder joint. That movement drove the first gear in a simulated gear train. Using distance from shoulder joint to wrist joint, the average diameter of the driving gear was mapped to the learner's body; when the learner altered the size of the physical spins, that action altered the size of the gear on screen in real time. Using the learner's real time wrist speed, the velocity of the gear spin was also mapped in real time. **Congruency means a large overlap between the action performed and content to be learned**. In that study, the learners that understood mechanical advantage (on a traditional test) also showed greater competency during gameplay, because they consistently chose the correct diameter gear during the virtual bike race. This is an example of how gesture can be part of both the learning situation and assessment.

#### Immersion/presence

Construct c has been called *sense of immersion* in previous articles describing the Johnson-Glenberg embodiment taxonomy for education (Johnson-Glenberg et al., 2014a; Johnson-Glenberg and Megowan-Romanowicz, 2017). Slater's lab posits that immersion is a non-subjective property of the technological system (which includes attributes like Field of View (FOV) and fidelity to environment). They distinguish between presence and immersion and state that presence is what is subjectively felt by the user, although they concede the two terms are “subjective correlates” (Slater and Sanchez-Vives, 2016). In America, researchers have tended to conflate these two terms. Slater and others (Witmer and Singer, 1998) assert that the two terms should be kept separate because presence is always a subjective experience and not as quantifiable as the immersivity of a system. But, the two terms are inextricably “tangled” (Alaraj et al., 2011), and given the high fidelity and immersive affordances of the current spate of VR technologies, it may be appropriate to assume the majority of users will be in high fidelity and highly immersive VR environments. As the amount of immersivity in the technology begins to asymptote, perhaps more weight should be placed on the construct of presence. This is not to say that VR is on the flat slope of modal innovation. There is much work to be done with haptics and olfaction, but the large amount of variance of immersivity seen in the systems of the early 2000's, has been attenuated. The levels of quality for optics, lag, and audition are sufficient for the majority of users to suspend disbelief and feel translocated.

The author proposes using the one term *presence* to also connote a very high degree of immersion, because the amount of immersion is universally high in current immersive VR. When discussing MR platforms, the immersivity distinction may still be relevant. To show how we mesh the two terms, the fusion term of *immersion/presence* will be used. Under the construct of immersion/presence, there are subsumed other factors that are critical to learning, e.g., motivation and prior knowledge, which are clearly important in learning. Many of those factors are not under the control of the lesson designers. One might experience low presence in a lesson if prior knowledge were extremely low and inadequate for the task^3^.

Several new taxonomies for embodiment are being proposed that do not include the third construct of “sense of immersion” or presence (Skulmowski and Rey, 2018). In many ways, a two axes model makes for a tidier taxonomy. However, we believe that to reframe the embodied taxonomy for education for 3D immersive VR, a construct for immersion/presence is crucial because presence is one of the unique and profound affordances of VR.

When learners experience high presence, they have suspended disbelief enough to engage meaningfully with the virtual. Players often report they lose some track of time and place. It is known that learning is facilitated by engagement and motivation (Csikszentmihalyi, 1997). If feeling presence connotes that the learner's body is in the virtual world, then higher presence might also correlate with higher levels of embodiment. The original, embodied taxonomy from Table 1 (Johnson-Glenberg and Megowan-Romanowicz, 2017) consisted of four delineated degrees along the continua of the three constructs. Reprinted table is open source from Johnson-Glenberg and Megowan-Romanowicz (2017).

**Table 1:** Construct magnitude within degrees in the Embodied Education Taxonomy.

**Degree**	**4th**	**3rd**	**3rd**	**3rd**	**2nd**	**2nd**	**2nd**	**1st**
**EMBODIMENT CONSTRUCT**								
Sensori-motor	H	H	H^*^	L	L	L	H^*^	L
Gestural congruency	H	H	L^*^	H	L	H	L^*^	L
Sense of Immersion	H	L	H	H	H	L	L	L

*H, High; L, Low*.

* *An ill-conceived, but possible configuration*.

Note that the cells with asterisks would be poor contenders for lesson design. Using a large gesture that is poorly mapped to the learning situation is not predicted to induce felicitous learning (e.g., moving one virtual electron in a magnetic field by performing three jumping jacks). It is kept in for the sake of symmetry, and, well, because bad lessons *do* happen.

### 3D figures for 3D constructs

**Figure 1:**
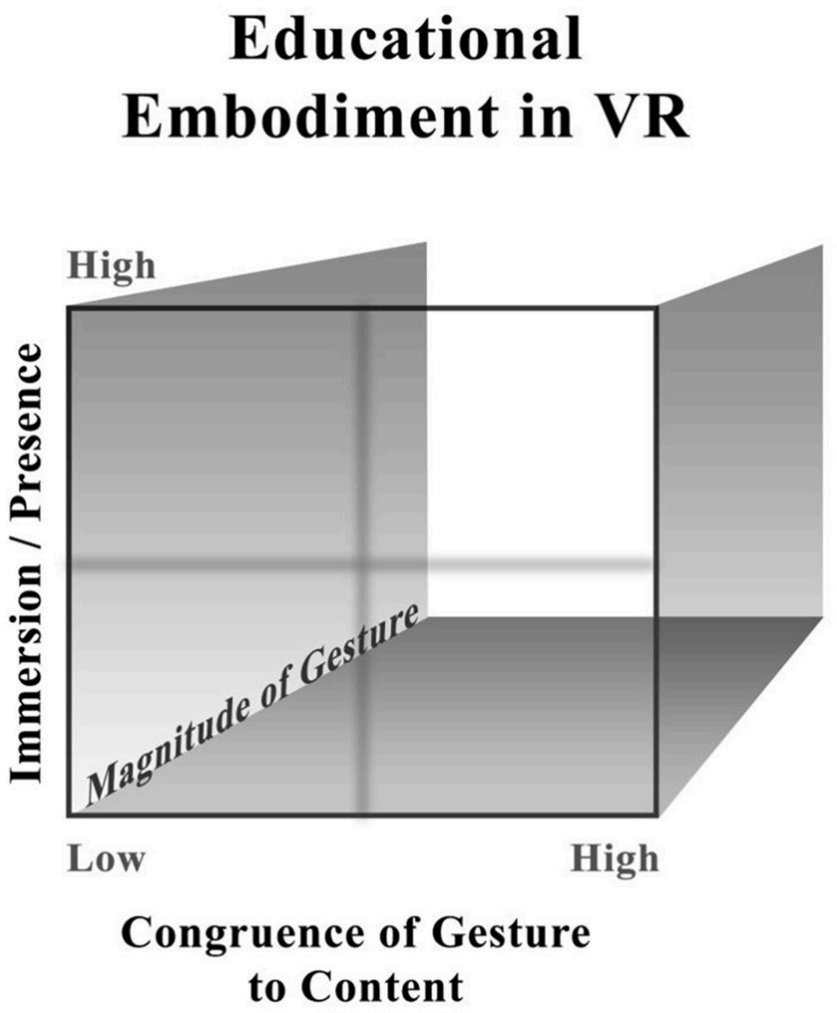
Cube of embodiment in educational VR content.

The new graphic in Figure 1 takes into account the continuous nature of the three constructs. It maintains the concept of immersion/presence. The crosshairs in the middle allow the reader the opportunity to partition the large space into more tractable low and high spaces; it could even be imagined as eight sub-cubes. It should be stated, that those who design multimedia lessons to be used in classrooms (as opposed to experimental labs) understand that lessons rarely fall neatly into any one sub-cube or bin. Because magnitude of the gesture (i.e., the amount of sensori-motor engagement) may prove to be the least predictive construct for content comprehension, it is relegated to the Z axis. The Z axis, or depth, is usually more difficult to conceptualize in a graphic. The goal for graphics like these is to aid researchers and designers in visualizing embodiment in educational content and aid the community in using the same terminology. These graphics should also spur researchers to assess the orthogonality of the constructs. RCT's on the three constructs and how they interact during learning are greatly needed.

## More on gesture and learning in 3D

The use of hand controls in VR has the potential to be a powerful catalyst for engaging students, heightening agency, and aiding in the comprehension of complex 3D concepts. The new hand controls are significantly more intuitive than traditional game consoles and the ease of entry has been remarked on by multiple users and designers, including in the Oculus Designer Best Practices Guidelines (Oculus, 2018). Hands and arms are untethered, multiple markers no longer need to be strapped to the body, and now more distal body parts (e.g., the feet) are being extrapolated with smaller peripherals. The era of immediate full body mapping will be shortly upon us. Until that time, we begin by focusing the embodiment lens on hand-based gestures.

There are four classic hand gestures that have been codified (McNeill, 1992). These gestures are: beat (usually moving the hand rhythmically with speech), deictic (pointing), iconic (i.e., a victory sign with the index and middle finger spread), and metaphoric (where the motion often serves as the metaphor). An example of a metaphoric gesture would be flipping a palm past the ear toward the shoulder to connote something that “was in the past.” In mixed and virtual reality environments iconic and metaphoric gestures are often meshed, e.g., in an educational evolution game built by our lab, butterflies are captured with a virtual hand-held net. Grabbing a hand control trigger makes the avatar hand into a fist on screen (used to grab the butterfly net–iconic) and swinging the hand makes the net *swish* (capturing the virtual butterflies upon collision–metaphoric). In the end, the iconic vs. metaphoric gesture distinction may not be very helpful in VR's dynamic and fantastical environments. This lab often uses the term representational gesture. The latest hand control model as of Summer 2018 included with a Standalone VR headset comes with a dozen preprogrammed iconic gestures (e.g., OK, peace V, Vulcan greeting, etc.).

Beyond iconic gestures with a human-looking hand, your avatar's hands can look like anything. Hands could resemble wingtips to fork tines, and they can manipulate anything, from quarks to galaxies. Gesturing with a human-looking hand may have special affordances that further increase the sense of agency. It is known that using hands to be in control of the action on screen can attenuate simulator sickness (Stanney and Hash, 1998). It has been shown that users quickly begin to treat their avatars as if they were their real bodies (Maister et al., 2015). This is further supported by research comparing virtual and real world instances of the classic Rubber Hand Illusion (IJsselsteijn et al., 2006).

Gesture has been researched in education for years and over a wide range of topics. Abrahamson researches mathematics and proportionalities (Abrahamson, 2009). Alibali and Nathan explore learning and teaching diverse math topics including equation solving, word-problem solving, algebraic and geometric concepts (Alibali and Nathan, 2012; Nathan et al., 2014). Congdon et al. (2017) showed that children as young as 3rd grade retain and generalize content from a math lesson better when they received instructions containing paired speech and gesture (as opposed to sequential speech and then gesture). In a mixed reality study on astronomy, students learned more about dynamic concepts with full body movements (Lindgren et al., 2016). Many mixed reality studies move beyond simple gesture and incorportate whole body movement. In a previous study reported in 2016, a randomized control trial (RCT) varied the amount of embodiment in a mixed reality system called *SMALLab* (Situated Multimedia Arts Learning Lab). College students were randomly assigned to three separate platforms that allowed for varying amounts of both motor activity and congruency (embodiment) (Johnson-Glenberg et al., 2016). The topic was centripetal force. The platforms were: (1) *SMALLab*, where learners could physically swing a tangible bob-type object on a string overhead, (2) Whiteboard, where learners could spin their arms in a circle to manipulate the virtual object, and (3) Desktop, where learners could spin the mouse in circles while seated. Within the three platforms, the amount of embodiment was either high or low. All six groups gained physics knowledge equally from pretest to immediate posttest; however, from posttest to 2 week follow-up, the level of embodiment in the lesson interacted significantly with time. That is, the participants in the higher embodiment conditions performed better on the generative knowledge test—regardless of platform. This supports the hypothesis that better retention of certain types of knowledge can be seen over time when more embodiment is present during the encoding phase.

Beyond the concept that gesture may aid in lightening the cognitive load (Goldin-Meadow et al., 2001), there are other theories addressing why gesture may aid in learning. One theory is that using gesture requires motor planning and this activates multiple simulations even before the action is taken. Hostetter and Alibali (2008) posit that gesture first requires a mental simulation before movement commences, at that time motor and premotor areas of the brain are activated in action-appropriate ways. This pre-action, covert state of imaging an action appears to stimulate the same collaries as the overt action i.e., motor cortex, the cerebellum, and basal ganglia (Jeannerod, 2001). The combination of planning and then performing may lead to more motor and pre-motor activity during encoding, which might lead to a stronger learning signal and memory trace.

The duality of the immersion/presence afforded by VR meshed with the new interfaces of the hand controllers allows for unique learning possibilities. In much of the past research on learning in VR (e.g., Gutierrez et al., 2007), the focus has been on the technology and short shrift has been given to learning pedagogies behind the lessons. This state of affairs prompted Fowler (2015) to title an article, *VR and learning: Where is the pedagogy?* (Fowler, 2015). Fowler calls for a design-for-learning perspective and urges readers to consider the “value or benefits that VR would add” to each particular learning experience. Designers and users of VR should be more aware of learning theories, so a short summary of some relevant educational theories that could be integrated in VR lessons is included.

## VR and education

Researching VR and education is confounded by the fact that many authors consider “virtual worlds” to be isomorphic to VR, thus searches promising meta-analysis research on VR, see Merchant et al. (2014) as an example, are not very helpful in 2018. There has been little work to date on education and immersive VR (also called IVR) (Blascovitch and Bailenson, 2011). Scholars have been asking for educational research for some time (Mikropoulos and Natsis, 2011), but the resources and affordable technologies were not readily available. Up until 2016, most of the literature on VR and education was based on proprietary VR software and hardware. Research labs, the military, or commercial companies had to create in-house products that were too expensive for public consumption. In 2016, two sets of high-end headsets with hand controllers (Oculus *Touch* and HTC *VIVE*) came to the market. Studies on gesture in VR are slowly coming to light^4^.

The use of VR in education is so new, and its affordances are of such a multitude, that design guidelines solely for education in VR have not yet been published. A meta-analysis commissioned by the US military (Dixon et al., 2009) found 400 documents that had the words “2D, 2.5D, and 3D applications for information visualization, display development, and guidelines for applications of dimensionality.” The search stopped in the year 2006. The study reports benefits when 3D technology was used to:

“convey qualitative information, provide a rapid overview, facilitate mission rehearsal, visualize network attack and physical access vulnerabilities, and aid route planning. … practicing telemanipulation skills with sensor augmentation and can provide realistic simulator training (piloting, aerial refueling, etc.).” p. 11.

Dixon et al. (2009) found few studies that, if they reported on human performance at all, were not tied to performance with specific equipment. Thus, the findings were somewhat narrow and non-generalizable. More recently, a well-regarded second edition of *3D User Interfaces* (Laviola et al., 2017) has been published, but it includes little mention of pedagogies for learning and less than one page on bi-manual control in VR. In these early days, trial and error plays an outsized role in design. Education researchers borrow heavily from the entertainment designers, who focus on engagement, and not necessarily on retention of content. The dearth of studies highlights the urgency for a set of guidelines for designing content that allows users to make appropriate choices in a spherical space. Below are short summaries of three education theories that lend themselves to creating gesture-controlled content in immersive VR.

### Constructivist learning theory

This theory builds off of Dewey's (1966) concept that education is driven by experience. Piaget (1997) further describes how a child's knowledge structures are built through exploratory interactions with the world. Constructivism emphasizes authentic interactions with the world that are consistent with knowledge students are expected to develop (Duffy and Jonassen, 1992). Environments such as VR can provide opportunities for learners to feel present in goal-driven, designed activities. The interactions that they have with artefacts and interactional systems in these environments should facilitate the construction of knowledge about the activities (Dede, 1995; Winn, 1999).

This is a theory article that ends with real world design advice to enhance classroom learning experiences, so further definitions of constructivism have been culled from a teacher's textbook (Woolfolk, 2007). The bolded text below has been added by this author to highlight components that VR is especially well-suited to address.

Per Woolfolk, common elements in the constructivist perspective:

1. Embed learning in complex, **realistic, and relevant** learning environments.
2. Provide social negotiation and shared responsibility.
3. Support **multiple perspectives and multiple representations** of content.
4. **Knowledge is constructed** (built upon)—the teaching approach should nurture the learner's self-awareness and understanding of ongoing construction.
5. Encourage **ownership** in learning. p. 348

Point 2 regarding social negotiation is important in education, but not highlighted because it is still expensive to implement multiuser, synchronized learning spaces. Educational instances of real-time, multi-user social negotiations in VR are probably years away (for an update on multi-user VR in education see Slater and Sanchez-Vives, 2016). A constructivist example in STEM in mixed reality is provided in the section called Example of an Embodied Lesson and Experiment. In scaffolded, virtual STEM environments, the learners start with simple models and interact to create more complex ones over time. Learners receive immediate feedback and know they are the agents manipulating the objects. They know they are in charge of the constructing. When a lesson is appropriately designed, with incrementally increasing difficultly, and includes evaluative, real-time feedback, then learners are encouraged to become more metacognitive. Learners become evaluative about their output. They can re-submit or reconstruct models multiple times. In this way, agency and ownership are encouraged. Active learning is especially important in the STEM domain where the majority of young STEM learners drop out over time (Waldrop, 2015).

### Guided inquiry

Inquiry refers to the collection of methods scientists use to study natural phenomena, to advance and test hypotheses, to subject hypotheses to reasoned analysis, and to use data to explain and justify assertions. Inquiry can be used to describe the ways students can investigate the world as scientists might. Students can propose and test ideas about how the world works, analyze findings, and make arguments from evidence to justify their assertions (Hofstein and Lunetta, 2003). *Guided* inquiry emerged in the late 1980's as an effective practice because it had been shown that free, exploratory learning, on its own, could lead to spurious hypotheses. Minimally guided instruction is “less effective and less efficient” (Kirschner et al., 2006), until one has a sufficient amount of prior knowledge. Students benefit from pedagogical supports that help them construct conceptual models, or knowledge structures (Megowan, 2007). Guided inquiry methods with technology are being developed to help students build, test, and deploy conceptual models of phenomena which cannot be directly observed. VR is poised to be an important tool in this domain. Guiding learners toward accurate deductions does not mean hand-holding. It means giving just enough information so that the final deduction is made by the student, in this manner the students takes ownership over what they have learned. Many believe that some cognitive effort is needed for learning “to stick”; these concepts are in line with the desirable difficulties literature (Bjork, 1994; Bjork and Linn, 2006), and levels of processing research.

### Embodied learning

Human cognition is deeply rooted in the body's interactions with the world and our systems of perception (Barsalou, 1999; Wilson, 2002; Glenberg et al., 2013). It follows that our processes of learning and understanding are shaped by the actions taken by our bodies, and there is evidence that body movement, such as gesture, can serve as a “cross-modal prime” to facilitate cognitive activity (e.g., lexical retrieval; Hostetter and Alibali, 2008). Several studies by Goldin-Meadow's group have shown a direct effect of gestures on learning (Goldin-Meadow, 2014). Recent research on embodied learning has focused on congruency (Segal, 2011; Johnson-Glenberg et al., 2014a), which posits an alignment of movements or body positioning (the body-based metaphor—see Lindgren's work) with specific learning domains (e.g., learning about centripetal force and circular motion by performing circular movements as opposed to operating a linear slider bar, Johnson-Glenberg et al., 2016). Virtual and mixed reality environments afford the opportunity to present designed opportunities for embodied interactions that elicit congruent actions and allow learners opportunities to reflect on embodied representations of their ideas (Lindgren and Johnson-Glenberg, 2013).

Embodied learning is probably most effective when it is *active*, and the learner is not passively viewing the content, or watching others interact with manipulables, as reported by Kontra et al. (2015). If the learner is induced to handle the physical content, or to manipulate the content on screen then they must be active and moving the body (which activates more sensori-motor areas). James and Swain (2010) placed 13 young participants (approximately six years of age) in an fMRI scanner. The children either actively manipulated an object (called a self-generated action) while hearing a new, novel label, or they watched an experimenter interact with the object. Motor areas of the participants' brains were more likely to be activated upon subsequent viewing when they self-generated the action, as opposed to observing it.

As highlighted earlier in this article, “embodied and embodiment” are evolving terms. Computer-mediated educational technologies are changing rapidly as well. The new VR hand controls will allow for active engagement and high levels of embodiment in lessons. Using virtual content, teachers will not be constrained by having to purchase specific physical manipulables. While haptics and mass are constructs that the virtual world does not yet easily accommodate, their absence should not be viewed as barriers to designing high quality, high embodied content. In-headset cameras can now capture articulated finger movements and this will lead to further advances and uses of naturalistic gestures. Given that gestures and embodiment may figure prominently in educational VR in the future, this article includes an example of a highly embodied lesson that was built for a mixed reality environment. The next section also cites effect sizes to aid researchers in future experimental and research design.

### Example of an embodied lesson and experiment

This section presents experimental evidence supporting the hypothesis that active and embodied learning in mediated educational environments results in significantly higher learning gains. Examples of types of gestures are discussed and new inferential statistics have been run on the data included in this article. There is currently a dearth of RCTs for VR in STEM education. Educational RCT's can be found in both mixed reality (Lindgren et al., 2016) and augmented reality (AR) environments (Squire and Klopfer, 2007; Dunleavy et al., 2009; Yoon et al., 2012). The results usually favor the experimental conditions, and the more embodied and/or augmented conditions.

The electric field study described in this section was conducted before the latest generation HMD's with hand controls were commercially available. Immersion was one of the goals and so a very large projection surface was used to induce some presence; however, because the real world was always present on the periphery, this should not be considered VR. This was an MR study using a whiteboard surface with a 78 inch (1.98 m) diagonal. This lab has researched in mixed and augmented reality spaces for science education for over a decade; the range of topics includes geography (Birchfield and Johnson-Glenberg, 2010), nutrition science (Johnson-Glenberg and Hekler, 2013; Johnson-Glenberg et al., 2014b), simple machines (Johnson-Glenberg et al., 2015), physics (Johnson-Glenberg et al., 2014a, 2016) and forces. The full article describing the electric field study and the seven learning tasks can be found at Johnson-Glenberg and Megowan-Romanowicz (2017).

When designing for complex science topics, care is always taken to scaffold both the number of elements onscreen and the amount of interactivity necessary to optimally interact with the user interface. For a history of scaffolding in the learning sciences, see Pea (2004). Designing to mitigate the effects of content difficulty and user's physical interactions requires a multidisciplinary approach, previous research has been published on multimedia design with 2D content (Sweller et al., 1998; Mayer, 2009). Many pitfalls of poor scaffolding can be avoided with multiple playtests that include naïve users (Johnson-Glenberg et al., 2014c). Whenever this lab has scrimped on playtesting, the end product has always suffered.

For the study, a 1 hour-long series of seven simulations was created to instruct in Coulomb's Law. The study did not start with the full equation, but built up to that somewhat complex equation. Each of the four variables in the equation was introduced one at a time, and participants had multiple exposures to, and interactive practice with, each variable. The first task in the seven task series refreshed the college students' knowledge on the topic of atoms and charge. The final task revolved around the conditions needed for a lightning strike. Individual videos on the tasks (and free, playable versions of most of the games) can be experienced at https://www.embodied-games.com.

### Design

The study was a 2 × 4 design, the first factor was time with two levels: pretest and posttest. The second factor was condition with four levels: (1) Control - Symbols and Text (S&T), (2) Low Embodied (where participants watched animations or simulations), (3) High Embodied, and 4) High Embodied-with Narrative. The final two conditions were high embodied because participants were able to physically interact with, and construct, models onscreen. In the high embodied conditions participants' gestures were gathered via the *Microsoft Kinect sensor*.

The study was carried out in accordance with the recommendations of U.S. Federal Regulations 45CFR46 under the guidance of a state university's research, integrity and assurance office. The protocol was approved by the Institutional Review Board (IRB). All participants were over 18 years of age and signed written informed consent in accordance with the Declaration of Helsinki. The college students were randomly assigned to condition. The first two conditions were considered passive because the learners' “hand grab” gestures only served to advance to the next screen. The final two conditions were considered active because multiple gestures using the hands, arms, and knees were used to manipulate the content on screen, as well as to advance the screens.

Throughout the lesson, multiple high embodied and gesturally congruent movements were used to facilitate learning. The example below details simulation number three (out of seven) that focused on vector comprehension. This task was chosen because it is an **example of a 2D lesson that might be more efficacious if translated to a 3D immersive VR environment** because the electric field surrounds us in three dimensions. High school and college students often do not understand the spherical nature of the electromagnetic field from 2D instructional texts and computer models (Megowan-Romanowicz, personal communication, December 4, 2017).

**Figure 2:**
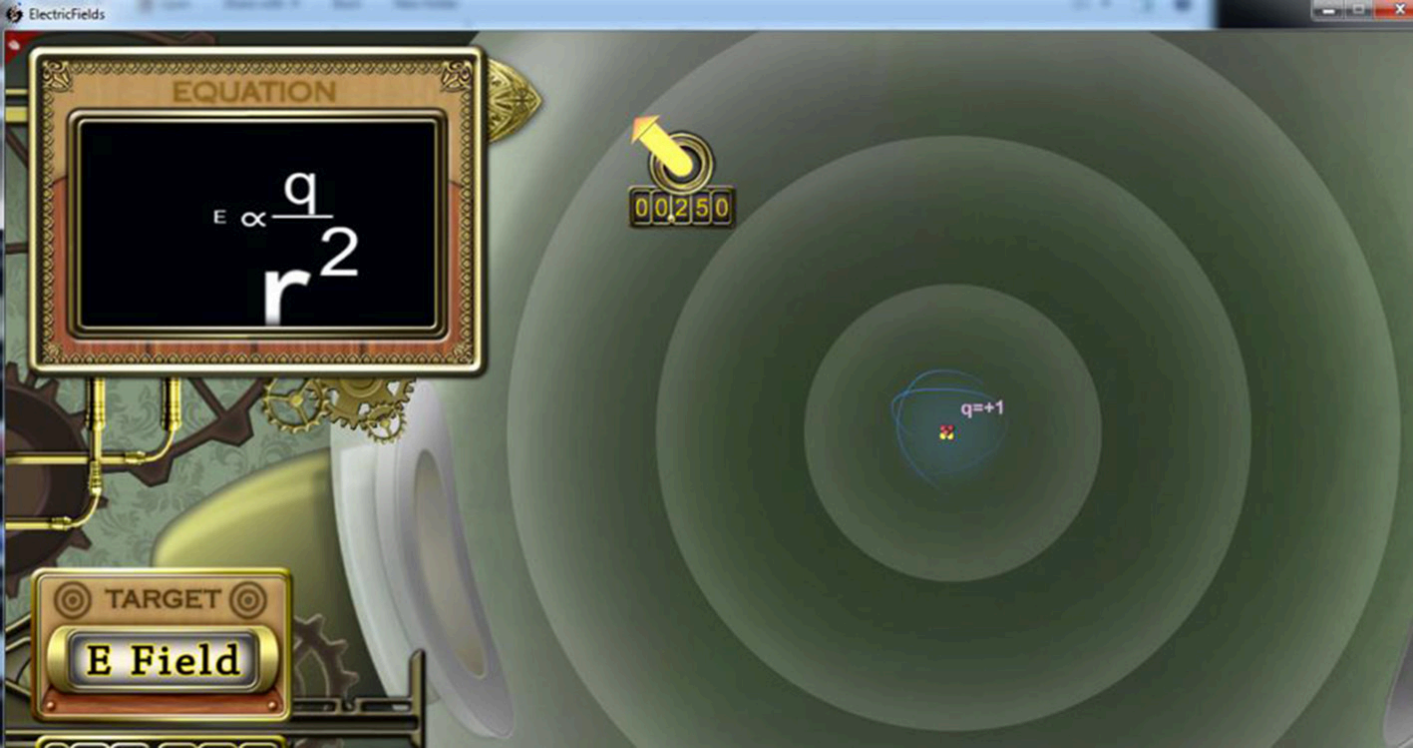
Screen capture of “Vector van Gogh” interactive task. Participants' gestures were captured with the *Kinect* sensor as they drew a vector (yellow arrow) in 3D space.

Figure 2 is a screen capture of simulation three called “Vector van Gogh” where participants were able to draw vectors. At the top left of the screen is a dynamic representation of a portion of Coulomb's Law. The symbols in this equation box (technically a “proportionality” since the constant *k* is missing) change in real time, such that, the size of the symbols represents the magnitude of each component. *E* is the electric field at a point in space, the numerator *q* represents the magnitude of the fixed charge in the center of the screen (+1). The denominator *r* represents radius and is squared. The radius is the distance of the free charge (the yellow circle) from the fixed charge in the middle of the screen. The fixed charge is represented by the tiny atom in the center. Designing with scaffolding means that the full proportionality for Coulomb's Law is not presented until the learner has been exposed to each variable separately (around simulation number 6).

**Figure 3:**
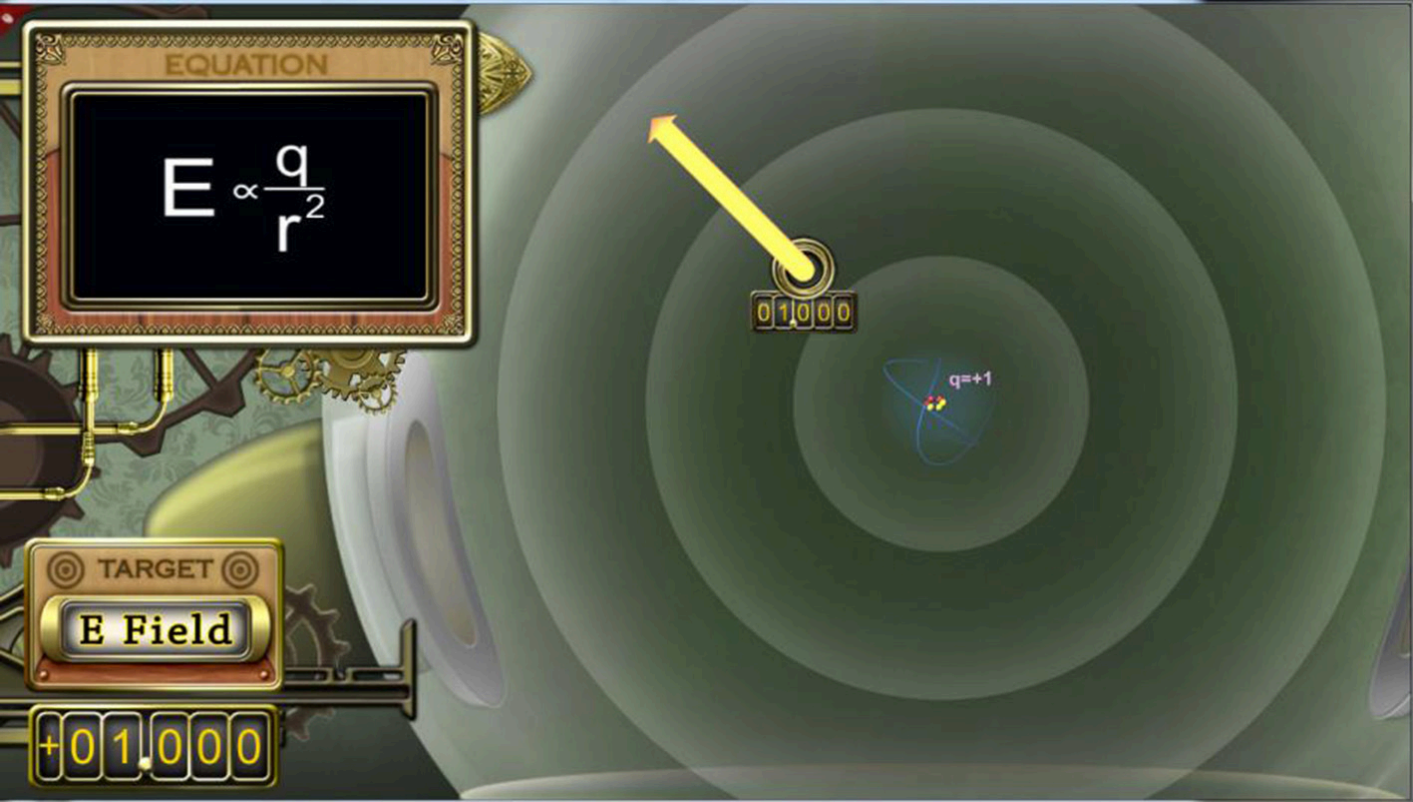
Screen capture of a later trial. The vector is longer and the dynamic equation for the electric field has changed as well. Note that *E* is much larger and the *r* is smaller (compared to Figure 2), these components change dynamically as the hand moves in real time.

In the high embodied conditions, the large arrow (yellow vector) is physically drawn by the participants. In the other conditions, participants either worked with symbols and text, or they passively watched animations of the yellow vectors being drawn over seven trials. The viewed animations included two errors, similar to what happened on average in the high embodied conditions. In the high embodied conditions, the *Kinect 360* sensor was used to track the right wrist joint. When the learner held down the clicker button, that signaled the start of the yellow vector—the tail would be set. The tail always began in the yellow circle (in Figure 3 the number 00.250 is shown under the start circle). The learner would then draw, via gesture, the vector's length and direction. With the release of the clicker button, the end of the vector (the tip) or arrow head would appear. If the learners were satisfied with the constructed virtual vector, they would hit submit. This constructed vector symbolized how the free particle would move when released. Thus, with larger or smaller embodied gestures, vectors of varying magnitudes were freely created by the learner with a swipe of the arm.

An algorithm was created to assess the quality of the submitted vector, comparing both its direction and length to an expert-drawn vector. If the learners' vectors were more than 5% discrepant, they had two more chances to redraw and resubmit. If the vector was still incorrect on the third try, the expert vector was displayed via animation and the next task appeared. In the equation box on the upper left corner of Figure 2, a relatively small electric field (*E*) at that point in space is shown. Note that the radius (*r*) is so large that it extends out of the equation box. That is because the free charge is far from the central, fixed charge (q = +1).

Figure 3 is a screenshot of a later trial in which the yellow start circle (aka free charge) is closer to the fixed central charge of +1. Again, the participant would draw an arrow to show the expected movement of the free charge. In this trial, the vector should be moving away from the positive fixed charge and it should be larger than the previously drawn one as the E Field is now 1.000. In the equation box, note how *E* is much larger and the *r* is smaller in size compared to Figure 2. This lesson reifies representational fluency in that the symbols map to the pictorial graphics, which in turn map in real time to the embodied movements of drawing.

The focus of this summary is on the gesturally passive vs. active conditions. For ease of interpretation between active and passive, the four groups have been collapsed into two.^5^

### Results of the electric field study

The study began with 166 participants. The four groups were matched at pretest and they remained matched when combined into two groups (*p* < 0.30). Two types of tests were administered, the first was a more verbal assessment that used a keyboard for typed responses to multiple choice and open-ended questions, in that assessment the two high embodied groups performed better, *M* = 49.9 (11.6), compared to the two passive groups (symbols+text and low embodied) *M* = 46.7 (13.1). The effect size or Cohen's *d* was small 0.22, but it favored the high embodied group.

**Figure 4:**
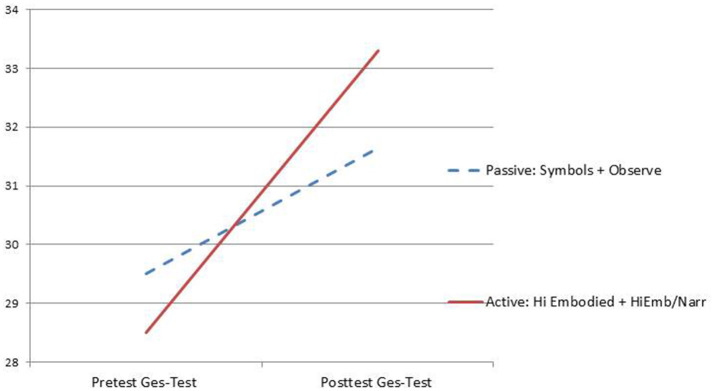
Graph of gains from the embodied vector comprehension test created using the *Wacom* tablet (The Ges-Test).

The second measure was an innovative gesture-based assessment, called the Ges-Test. This was created to allow participants to construct vectors by free hand drawing. Participants moved their fingertip along a large touchpad called the *Wacom*™ *Intuous Pro* (15.1 inch or 38.4 centimeter drawing diagonal). This allowed the participants the ability to speed up or slow down their movements so that the concepts of positive and negative acceleration could be assessed. Eight questions were analyzed. The hypothesis was that the gesture test would be more sensitive to revealing learning gains that might be attributed to embodiment during the encoding intervention phase. On the Ges-Test the active and embodied groups performed significantly better than the passive groups on the posttest, *F*_(1, 132)_ = 3.77, *p* < 0.05. See Figure 4.

### Study Conclusion

These results support the hypothesis that when learners perform actions with agency and can manipulate content during learning, they are able to learn even very abstract content better than those who learn in a more passive and low embodied manner. When tested with gesture on the topic of vectors and motion, the high embodied students showed they learned more. Given that being active and using congruent gestures seems to facilitate learning, we support designing VR content that makes use of the new VR hand controls for both learning and assessment purposes. Creating assessments that use gestures mapped to the hand controls locations in 3D space seems a productive path forward.

## Prudent VR guidelines thus far

For the most part, immersive VR educational lessons and studies have occurred primarily in adult populations (Freina and Ott, 2015). These have occurred in a variety of fields from medicine, e.g., intricate maneuvers involved in craniofacial repairs, (Mitchell et al., 2015), to behavioral change interventions, for solid examples see the innovative work on PTSD reduction by Rizzo et al. (2010). A chapter by Bailey and Bailenson (2017) provides a speculative overview of how VR might affect youth and cognitive development, but longitudinal effects of VR exposure are unknown at this point. Because so little is known about youth and VR, the guidelines included at the end of this article are recommended for players 13 years and older (similar to the constraints and advisements seen on the most popular commercial headsets).

In terms of education and classroom adoption, the first iteration of affordable VR for entire classrooms has been with mobile. Exploratory results have been reported using systems such as *Google Expeditions* (Minocha et al., 2017). Innovative work is also being done with MR goggle experiences in museums and at some historical cites (an example from Knossos is described by Zikas et al., 2016). One prediction is that when the Standalone headsets, which do not require phones or separate CPU's, become available, then immersive VR experiences with a hand controller will become more popular for classroom use. When VR becomes affordable, educators will be in need of quality content. What will high quality pedagogical VR look like? Should everything 2D just be converted to 3D? We agree with Bailenson who posits that VR should be used in instances where it is most advantageous (Bailenson, 2016). He lists four:

- **Impossible**—E.g., you cannot change skin color easily, but in VR you can inhabit avatars with different skin colors with profound results (Banakou et al., 2016; Hasler et al., 2017). You cannot perceive a photon going directly into your eye in the classroom, but in the next section we describe a VR simulation doing just that.
- **Expensive**—You cannot easily fly your whole school to Machu Picchu.
- **Dangerous**—You would not want to want to train emergency landings by crashing real airplanes.
- **Counterproductive**—You should not cut down an entire forest to instruct on the problems of deforestation.

When designing for VR for education, Dalgarno and Lee presciently published several affordances for three dimensional VR environments, which they call VLE's (virtual learning environments) (Dalgarno and Lee, 2010). The five listed below pertain to both PC-based 3D worlds and immersive VR (as this article uses the term). This author's notes are in brackets.

Affordance 1: Use VLE's to facilitate learning tasks that lead to the development of **enhanced spatial knowledge representation** of the explored domain. [This is in-line with this lab's sentiments that 3D and the affordances of spatial reasoning represent a profound affordance of the technology. If no special insights will be gained from using the more costly VR equipment, then stick with 2D models].
Affordance 2: Use VLE's to facilitate experiential learning tasks that would be impractical or impossible to undertake in the real world. [This is similar to Balienson's tenet.]
Affordance 3: Use VLE's to facilitate learning tasks that lead to **increased intrinsic motivation and engagement**. [The research community suspects that VR, regardless of the quality, will continue to enhance engagement, which has been shown to increase learning. However, one further prediction of ours is that the novelty and heightened engagement will wane over multiple exposures, and at that time quality pedagogy will be driving the learning. Tightly controlled RCTs have yet to be performed on these issues. There have been several early studies comparing learning in a 3D headset to viewing the content on a computer monitor screen as the control condition (Gutierrez et al., 2007), but it is time to move beyond simple 2D PC comparisons.]
Affordance 4. Use VLE's to facilitate learning tasks that lead to improved transfer of knowledge and skills to real situations through **real world contextualization of learning**.

“Specifically, because 3-D technologies can provide levels of visual or sensory realism and interactivity consistent with the real world, ideas learnt within a 3-D VE should be more readily recalled and applied within the corresponding real environment.” p. 22.

Affordance 5: Use VLE's to lead to richer and/or more effective collaborative learning as well as richer online identity construction and a **greater sense of co-presence that will bring about more effective collaborative learning**. [This rings true as well, although we note that a zero-lag, multi-user classroom experience may still be a few years away.]

### A high embodied VR lesson using hand controllers

Deftly meshing education with games is a far trickier business than one might suspect. This author has been building multimedia educational content for over two decades and can admit to creating several epically flawed “edu-games” in that time. Unfortunately, the majority of education apps available today for free are still neither highly educational nor sustainably entertaining. Education is underfunded for the sort of iterating (with quality graphics included) needed to create compelling and effective learning games. Education game designers often take their cues from entertainment game designers, for better or worse. As VR comes of age, the first popular titles are going to be the entertainment ones. Quality education games will come later. One prominent game creator giving advice on VR design is Jesse Schell. His Oculus 2 Conference presentation (2016, https://www.youtube.com/watch?v=LYMtUcJsrNU) contains many design nuggets. These range from the broad: Keep the horizon level; Proprioceptive disconnect is bad, i.e., you should not be a reclining human with a walking avatar body; Sound is vital and takes twice as long to get right in VR. To the specific: 3D with 9DOF is well-suited for peering into multidimensional objects like brains and engines, however, if you lock an object near the user's POV then you need to give the object a bobbing motion or users will assume the system is frozen.

The educational VR community does not yet have a set of guidelines for how to implement hand controls and gesture for embodied education. Before ending with a list of design guidelines for that space, a VR lesson is described that incorporates these guidelines. We consulted on creating an Alpha version of a high school-level chemistry lesson in a VR open world called *Hypatia*. The premise of *Hypatia* is that it is a multi-player world primarily built for social entertainment. One of the company's mantras was “never break immersion.” But, learning scientists know it is also important to build in time for reflection during a lesson so that students can create meaning around the intense stimuli. Never break immersion may be a guideline from entertainment that does not migrate well into the education community. In a goal-driven learning situation it may be desirable to bring learners out of the experience, perhaps to a virtual whiteboard. It may be efficacious to request learners remove the headset to make handwritten notes, or perhaps engage in face-to-face collaborations/questioning with a partner before resuming the immersive experience. These are empirical questions.

In the multi-player virtual world called *Hypatia*, players first create non-human avatars with pre-populated parts. The early module described here was called Kapow Lake; it was conceived of as a high school chemistry and physics lesson using fireworks as the topic. Two learning goals were embedded: (1) understand which metal salts burst into which colors, and (2) understand the elementary physics behind why the burst is perceived as a particular color.

**Figure 5:**
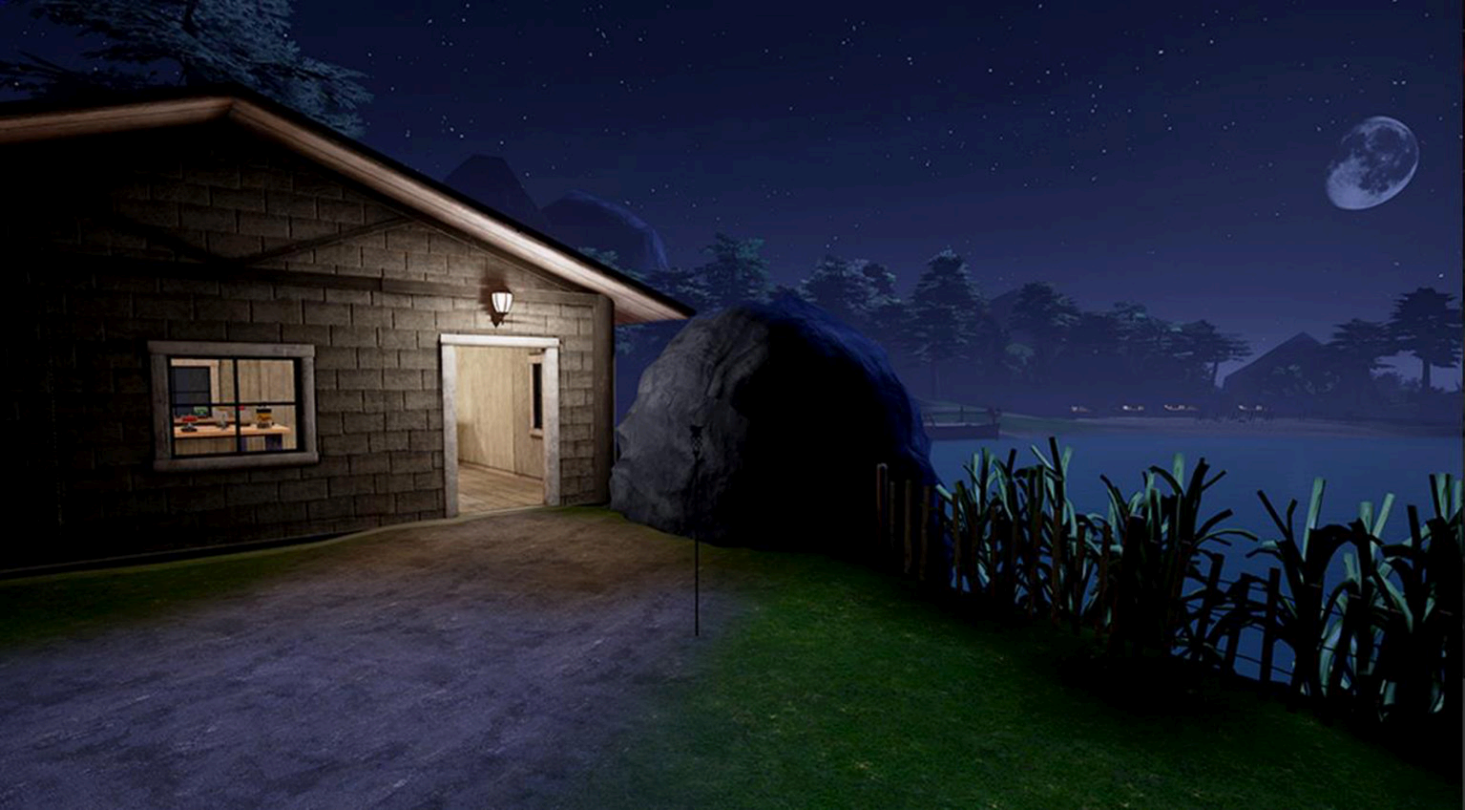
Screen capture from the chemistry lesson in *Hypatia*. Note signposting via the lit door to encourage learners to enter the experimentation shed.

Players start on the beginner side of the lake, they can watch fireworks in the sky and are motivated to build some of their own. Using light cues, we “signpost” players via the lit doorway to enter the experimentation shed. See Figure 5.

**Figure 6:**
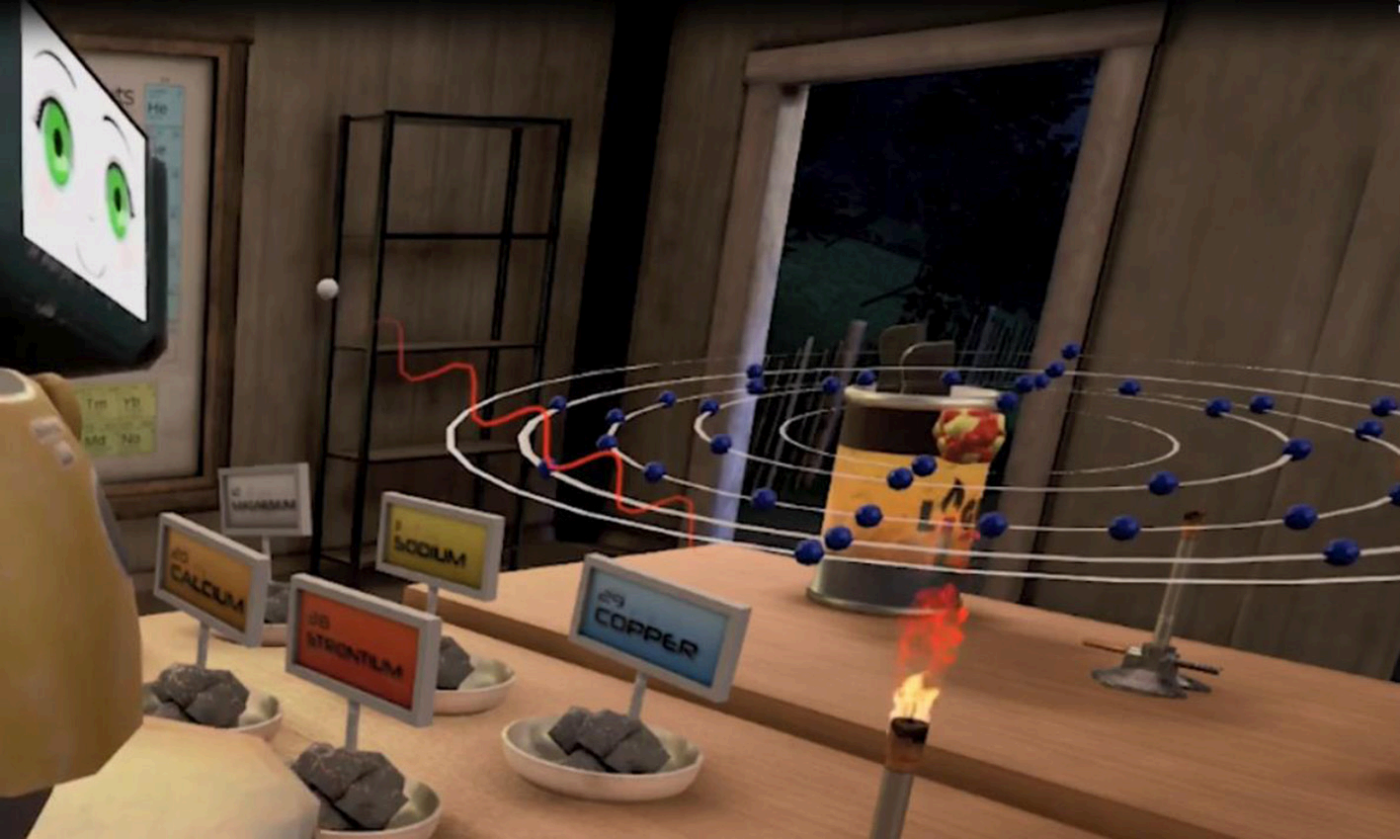
As the strontium electron moves back to its stable orbit, a photon is released that is perceived to be in the red spectrum (from *Hypatia*).

Theories of constructivism and guided exploration are prevalent throughout the lesson. In order to construct their own fireworks players must first master the names of the colors. Players would grasp the triggers of the hand controls (i.e., *HTC Vive*) and when the avatar hand collided with a metal salt, the salt would be picked up. The first series of gray metal salts (see Figure 6) did not have the colors on the labels. So players did not know that the salt called strontium would burn red. Via systematic exploration, they would place each salt into the flame of the Bunsen burner and note the color that the salt burned.

Figure 6 shows the avatar (Jessica) on the left side of the screenshot. The salt labels are now colored and visible (i.e., if strontium burns red, how will copper burn?). This lesson takes advantage of several of the affordances associated with VR, one of which is making the “unseen be seen”, now an individual atom of strontium can be shown. After Jessica places the gray salt over the flame a Bohr atom model of strontium appears on top of the flame.

Another of the profound affordances of VR is the immersion into three dimensions. Note that the screenshot is taken from the 3rd person POV for the purposes of edification, but Jessica, the human player, is seeing the atom floating above the burner in 1st person or a “head on” POV. After she places the strontium over the heat, the outer electron moves out of the stable outer orbit. The unstable orbit is shown briefly as a dotted ring during play. Quickly, the electron falls back to its more stable orbit, as it does this a packet of energy called a photon is released. This photon is perceived in the red spectrum. In Figure 6, the photon has been visualized as *both* a red wave and a particle heading toward the eye. Jessica is watching the dynamic model in 3D and she perceives the photon as traveling directly into her eye. (This is perhaps the only thing humans want heading directly toward our eyes!) The sinusoidal movement was designed to be somewhat slow, so it would not be alarming.

**Figure 7:**
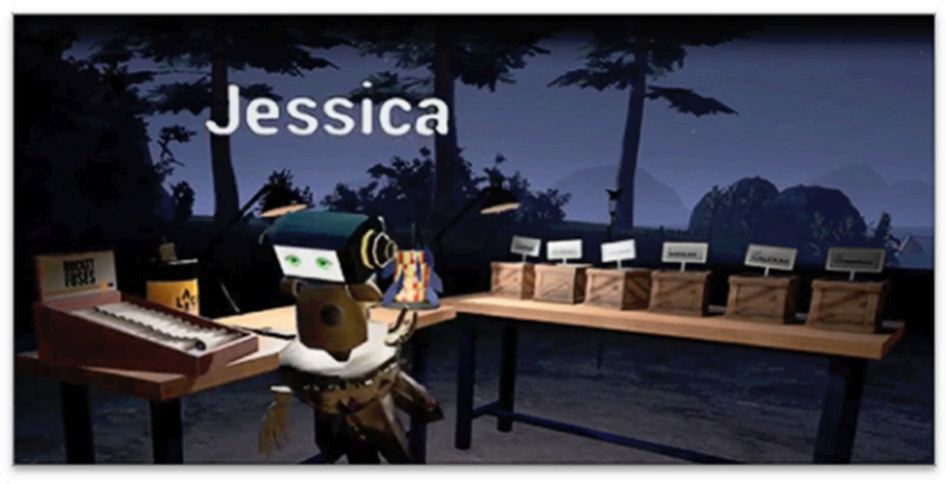
The learner is now able to construct multi-stage rockets. Note the base of the firework on the table behind the avatar (from *Hypatia*).

The simulation of the photon as a wave reifies the concepts that energy is released by the heat burst, and that that the energy is then perceived by the human eye as a visible wavelength. The five other salts release electrons from different orbits, thus creating different wavelengths. Once players are able to match all six metal salts to their colors, they are signposted to exit the back door to the expert's multi-staging fireworks area (Figure 7).

Players are allowed several minutes of free exploration to construct rockets. If they are not building functional ones in the time limit, then we again signpost (via object blinking) the sequential procedures for construction (e.g., tube first, then fins, salts, fuse, then the cone top). After two correct constructions, players are instructed, via text in the headset, to build multi-stage rockets with very specific sequences of colors. This is an engaging task, but it also serves as a form of stealth assessment (Shute, 2011). Now a teacher or spectator can observe whether the student really understands how strontium and copper need to be sequenced to make a red then a blue explosion.

### Design principles for embodied education in VR

The new VR principles are grouped first as general guidelines and second as those pertaining to gesture and hand controls. They are listed in the order that they are often performed in. That is, a design and development team starts with a paper version of the interface. It is necessary though to iterate on a module several times before the module is ready for release. It will never be perfect; strive for 80% satisfaction. In an effort to keep the number of guidelines tractable, the article closes with the *Necessary Nine*. An important point to drive home for designers of education in VR is to remember that presence is immediate and for the learners to internally adjust to that feeling, it can take time. VR for entertainment can purposefully overwhelm, but the goal of education is for learners to *leave the space with new concepts embedded in their ever-changing knowledge structures* (the definition of learning). Some of your learners will also come to the task with low spatial abilities, and those students learn differently in 3D space (Jang et al., 2016). This is why the first start screen should always be somewhat sparse with a user-controlled start button. They can start when they feel acclimated. Declutter the user interface (UI) as much as possible, especially in the early minutes of the game.

### General guidelines

- Assume every learner is a **VR newbie**
  ◦ Not everyone will know the controls. Not everyone knows to look around. Users are now in a sphere and sometimes need to be induced to turn their heads… but only so far. Do not place important UI components or actionable content too far from each other. E.g., do not capture butterfly #1 at 10° and then force them to capture butterfly #2 at 190°. Be gentle with users' proprioceptive systems (where the body is in space). If the content includes varying levels of difficulty, allow the user to choose the level at the start menu. This also gives a sense of agency. This “start slow” advice comes from years of designing educational content.
- Introduce **User Interface (UI) components judiciously, fewer is better**
  ◦ When users build the first fireworks in our chemistry lesson, they can only make one stage rockets. The multi-chambered cylinders are not available in the interface until users show mastery of the simpler content. (Johnson-Glenberg et al., 2014c).
- **Scaffold** – also introduce **cognitive steps one at a time**
  ◦ Build up to complexity (Pea, 2004). As described in the electric field lesson instructing in Coulomb's Law, each component or variable in the equation is revealed one component at a time. Users explore and master each component in successive mini-lessons (Johnson-Glenberg and Megowan-Romanowicz, 2017).
- **Co–design with teachers**
  ◦ Co-design means early and with on-going consultations. Let the teachers, Subject Matter Experts (SMEs), or clients play the lesson/game at mid and end stages as well. Playtesting is a crucial part of the design process. Write down all comments made while in the game. Especially note where users seem perplexed, those are usually the breakpoints. Working with teachers will also ensure that your content is properly *contextualized* (Dalgarno and Lee, 2010), that it has relevance to and is generalizable to the real world once users are out of the headset.
- **Use guided exploration**
  ◦ Some free exploration can be useful in the first few minutes for accommodation and to incite curiosity, but once the structured part of the lesson begins, guide the learner. You can guide using constructs like pacing, signposting, blinking objects, etc. To understand why free exploration has not held up well in STEM education, see Kirschner et al. (2006).
- **Minimize text reading**
  ◦ Rely on informative graphics or mini-animations whenever possible. Prolonged text decoding in VR headsets causes a special sort of strain on the eyes, perhaps due to the eyes' vergence-accomodation conflict, but see Hoffman et al. (2008). In our VR game on evolution we do not make players read lengthy paragraphs on how butterflies emerge during chrysalis, instead a short cut-scene animation of butterflies emerging from cocoons is displayed.
- **Build for low stakes errors** early on
  ◦ Learling often requires errors to be made and learning is facilitated by some amount of cognitive effortfulness. In our recent evolution game, the player must deduce which butterflies are poisonous, just like a natural predator must. In the first level, the first few butterflies on screen are poisonous. Eating them is erroneous and depletes the learner's health score, but there is no other way to discern toxic from non-toxic without feedback on both types. Thus, some false alarms must be made. Later in the game, errors are weighted heavier. See recent learning from errors literature in psychology (Metcalfe, 2017).
- **Playtest often** with novices and end-users
  ◦ It is crucial that you playtest with multiple waves of age-appropriate learners for feedback. This is different from co-designing with teachers. Playtesting with developers does *not* count. Our brains learn to reinterpret visual anomalies that previously induced discomfort, and user movements become more stable and efficient over time (Oculus, 2018). Developers spend many hours in VR and they physiologically respond differently than your end-users will.
- Give players unobtrusive, **immediate, and actionable feedback**
  ◦ This does not mean *constant* feedback (Shute, 2008). Feedback and adjustments must be integrated into the learner's ongoing mental model, that process takes time.
- Design in **opportunities for reflection** (it should not be all action!)
  ◦ All educators/designers are currently experimenting with how to do this in VR. Higher level learning (cognitive change) is not facilitated by twitch. Reflection allows the mental model to cohere. Should the user stay in the headset or not? How taboo is it to break immersion? Should short quizzes be embedded to induce a retest effect (Karpicke and Roediger, 2008)? Dyads could ask each other questions? At this stage, it is advised that reflection should be incorporated, but we need more research on optimal practices within the headset.
- Encourage **collaborative interactions**
  ◦ Synced, multiplayer is still expensive, but it is a worthy goal. Try to include workarounds to make the experience more social and collaborative, either with a preprogrammed non-player character (NPC), having a not-in-headset partner interact via the 2D computer screen, or by designing sequential tasks that require back-and-forth in an asynchronous manner. A classroom collaboration and cooperation classic is Johnson and Johnson (1991).

### Using hand controls/gestures

This section focuses on using the hand controllers in VR for learning.

- Use the hand controls to **make the learners be “active”**
  ◦ Incorporate into lessons opportunities for learners to make physical decisions about the placement of content and to use representational gestures. Active learning has been shown to increase STEM grades by up to 20% (Waldrop, 2015).
- How can a **body-based metaphor** be applied?
  ◦ Be creative about ways to get kinesthetics or body actions into the lesson. E.g., if information is going to be displayed as a bar chart, first ask users to swipe upwards and make a prediction about how high one of the bars should go. Note: prediction is a metacognitive, well-researched comprehension strategy (Palinscar and Brown, 1984).
- **Congruency**
  ◦ The gesture/action should be congruent, i.e., it should be well-mapped, to the content being learned (Black et al., 2012; Johnson-Glenberg and Megowan-Romanowicz, 2017). For example, the action to start a gear train spinning should be moving something in a circle, not pushing a toggle up or down.
- **Actions strengthen motor circuits and memory traces**
  ◦ Performing actions stimulates the motor system and appears to also strengthen memory traces associated with newly learned concepts. See section entitled Embodiment for multiple citations.
- **Ownership and Agency**
  ◦ Gestural control gives learners more ownership of and agency over the lesson. Agency has positive emotional affects associated with learning. With the use of VR hand controls, the ability to manipulate content and interactively navigate appears to also attenuate effects of motion sickness (Stanney and Hash, 1998).
- **Gesture as assessment—Both formative and summative**
  ◦ Design in gestures that reveal the state of the learner's mental model, both *during learning* (called formative or in-process) and *after the act of learning* (called summative). For example, prompt the learner to demonstrate negative acceleration with the swipe of a hand controller. Does the controller speed up or slow down over time? Can the learner match certain target rates? This is an embodied method to assess comprehension that includes the added benefit of reducing guess rates associated with the traditional text-based multiple choice format. For an example, see the vector-based Ges-Test in Johnson-Glenberg and Megowan-Romanowicz (2017).
- **Personalized, more adaptive learning**
  ◦ Make the content level match the user's comprehension state – or be a little beyond the user's skill zone, as in Vygotsky's ZPD. Gesture research on younger children shows they sometimes gesture knowledge before they can verbally state it. Gesture-speech mismatches can reveal a type of readiness to learn (Goldin-Meadow, 1997). Thus, gestures can also be used as inputs in adaptive learning algorithms. Adding adaptivity (dynamic branching) to lessons is more costly, but it is considered one of the best practices in educational technology (Kalyuga, 2009).

## Conclusion

This article focuses on the two profound affordances associated with VR for educational purposes: (1) the sensation of presence, and (2) the embodied affordances of gesture in a three dimensional learning space. VR headsets with hand controls allow for creative, kinesthetic manipulation of content, these movements and gestures have been shown to have positive effects on learning. A new graphic “cube” is introduced to help visualize the amount of embodiment in immersive educational lessons. As more sophisticated extrapolation algorithms are being designed, the whole body can be mapped while in a headset. The mapping of full body movement may provide for even more creative gestures and actions for learning in 3D.

We encourage designers to also incorporate seamless assessment within VR lessons, perhaps using the idea of leveling up during learning. This would add adaptivity to the system, and gesture can be one of the variables that feeds the adaptive algorithm. Lessons should get more complex as the learner demonstrates competency on previous material. We also encourage designers to include collaboration, which will become easier when multiple players can be synced in the virtual space.

As the technology moves forward, designers should keep principles of best practices in mind, and instructors should consult the principles to help make instructional and purchasing decisions. The previous section describes 18 principles in more detail. This article ends with the top contenders below. If there are only resources to focus on a subset, then the author recommends the *Necessary Nine*.

- Scaffold cognitive effort (and the interface) - one step at a time
- Use guided exploration
- Give immediate, actionable feedback
- Playtest often - with correct group
- Build in opportunities for reflection
- Use the hand controls for active, body-based learning
- Integrate gestures that map to the content to be learned
- Gestures are worth the time - they promote learning, agency, and attenuate simulator sickness
- Embed gesture as a form of assessment, both during and after the lesson.

## Author contributions

The author confirms being the sole contributor of this work and approved it for publication.

### Conflict of interest statement

MJ-G also oversees the website called www.embodied-games.com. All education games on the site are free to the public as they have primarily been grant funded. Source code is available upon request.

## Footnotes

^1^Stanney and Hash (1998) conducted a three-way RCT with hand controls: passive, a mixture condition, and an active condition. In the active condition, participants had full control over movement in the VR space including pitch and roll, movements which were not needed for several of the, admittedly simplistic, tasks. They found that fewer symptoms of simulator sickness were reported in the mixture condition. Thus, targeted control was best.

^2^If cognition is conceived of as inputs and outputs interleaving with internal and external states, what happens during times of conflict? How do we resolve when the internal state does not match the external state? Perceptual Control Theory (PCT) (Bourbon and Powers, 1999) is framed in terms of the organism and the environment's interconnected system. There are inputs that determine the organism's actions, which again determine the input states. Thus, “there are two simultaneous relationships: (a) an observation that stimulus inputs depend on an interaction between behavioral outputs and independent events in the environment, and (b) a conjecture that behavioral outputs depend on an interaction between actual stimulus inputs (as perceived, not necessarily as they really are)…” p. 446. The phrase “as perceived” is important, because the topic is VR. Some is known about what happens physiologically when there is a mismatch between reality (e.g., the state of the inner ear) and a player's visual perception (e.g., flying swiftly through the clouds), and the result is usually simulator sickness. There is an eagerness to explore what happens in terms of long term cognitive change (i.e., “learning”) when the mismatch continues over time. Future theories should further explore what happens when we place people in fantastic, fully immersive environments that are perceived of as very real. In the upcoming years, our community needs to hone in the most applicable theories and run RCT's to verify best pedagogies for teaching with VR technologies.

^3^Wasted cognitive effort and emotional frustration would attenuate a sense of presence if the learner never ever understood which size gear to use to get the virtual bike up the virtual hill in the first place. Indeed, a handful of middle school players in the gears game called *Tour de Force* would insist on using the largest gear and spin furiously while the bike stayed in one place on the steep hill and the timer ran down (Johnson-Glenberg et al., 2015).

^4^One creative, full-body experiment assessed learning on the topic of microgravity. The experimental group (suspended in the VR immersion rig) answered one (out of two) key questions significantly better than the untreated control group. The control group was asked to merely imagine how long it would take to reach a door in a microgravity environment (Tamaddon and Stiefs, 2017). Clearly, larger studies with more robust assessments are needed.

^5^Videos simulations can also be seen at https://www.youtube.com/watch?v=eap7vQbMbWQ.
